# Pharmacotherapy and group cognitive behavioral therapy enhance follow-up treatment duration in gambling disorder patients

**DOI:** 10.1186/s12991-016-0107-1

**Published:** 2016-08-12

**Authors:** Sam-Wook Choi, Young-Chul Shin, HyunChul Youn, Se-Won Lim, Juwon Ha

**Affiliations:** 1 Korea Institute on Behavioral Addictions, True Mind Clinic, F7, KR Tower, 1 141, Teheran-ro, Gangnam-gu, Seoul, 06132 South Korea; 2 Healthcare and Information Research Institute, Namseoul University, 91, Daehak-ro, Seonghwan-eup, Seobuk-gu, Cheonan-si, Chungcheongnam-do 31021 South Korea; 3 Department of Psychiatry, Kangbuk Samsung Hospital, Sungkyunkwan University School of Medicine, 29, Saemunan-ro, Jongno-gu, Seoul, 03181 South Korea; 4 Chungmugong Leadership Center, Naval Education and Training Command, Republic of Korea Navy, P.O. Box 211, 111, Jinhui-ro, Jinhae-gu, Changwon-si, Gyeongsangnam-do 51655 South Korea; 5 Yonsei Forest Mental Health Clinic, Metroplaza 601, Jinkwan 2-ro 15-46, Eunpyeong-gu, Seoul, 03306 South Korea

**Keywords:** Gambling disorder, Treatment duration, Group cognitive behavioral therapy (CBT), Pharmacotherapy, Individual psychotherapy

## Abstract

**Background:**

Longer treatment duration is important for the successful treatment of gambling disorder (GD). This retrospective study investigated the factors and interventions that might enhance treatment duration in GD patients in South Korea.

**Methods:**

A total of 758 outpatients with a primary diagnosis of GD, who were treated in a clinical practice from 2002 to 2011, were assessed by retrospective chart review. We compared the treatment duration according to pharmacotherapy and group cognitive behavioral therapy (CBT).

**Results:**

Pharmacotherapy contributed to a longer duration of treatment maintenance, despite the patients’ gambling severity (p < 0.001). Participation in group CBT (p < 0.001) and antidepressants (p = 0.009) were associated with a longer treatment duration after adjusting for age, depression, and gambling severity. The treatment maintenance duration was the longest in those receiving combined antidepressant pharmacotherapy and group CBT (F = 35.79, p < 0.001).

**Conclusions:**

Group CBT and antidepressants seem to enhance treatment follow-up duration in GD patients. Additional studies are needed to advance GD prevention and treatment strategies.

## Background

Gambling disorder (GD), or pathological gambling, has rarely been studied as a disease. Until the late 1990s, little was known about the history and etiology of GD [1]. Recently, GD was classified as a clinically significant addictive disorder [2]. Although GD affects 0.2–5.3 % of adults and often results in severe damage to the lives of patients and their families, less than 10 % of those with GD receive professional treatment [3].

Cognitive behavioral therapy (CBT), 12-step programs, and pharmacological approaches have been proven to be effective treatments for GD [4]. The relative efficacy of medications remains a topic of ongoing research, as GD has only recently been recognized as a biological disease [5]. In recent years, several controlled clinical trials have been conducted on a variety of pharmaceutical classes and have established an evidence-based background for the disease [6]. However, the influence of these treatment modalities on treatment duration and adherence in GD has rarely been studied, and has not yet been revealed [7].

GD has been characterized as a chronic relapsing disorder with high treatment dropout rates, ranging from 43 to 80 % according to the study design including treatment duration and modalities [8]. One study reported an 18-month treatment duration; however, this study included only the participants who remained in treatment for a minimum of 60 days [9]. Thus, motivation and adherence to treatment are the most important factors in the successful treatment of GD [10, 11]. The majority of pathological gamblers do not seek treatment although they have serious psychosocial problems. Rather, family members pressure the affected relative to start and continue therapy [12]. Patients with addictive disorders often discontinue treatment before remission or recovery. Although dropout is an important factor in the outcomes of managing mental health problems [13], the clinical characteristics or interventions that might enhance the duration of treatment maintenance have not yet been revealed.

The current study investigated whether treatment intervention, and which treatment approach, is related to a lower rate of dropout in GD patients in South Korea.

## Methods

### Participants

The study participants were adults, aged 18–65 years, who visited the gambling clinic in Kangbuk Samsung Hospital between May 2002 and December 2011. From February 2012 to March 2013, we retrospectively reviewed the medical charts of these patients with GD.

Using the Structured Clinical Interview for the Diagnostic and Statistical Manual of Mental Disorders, 4th Edition (DSM-IV) for Axis I disorders (SCID-I), a tool for diagnosing GD and identifying psychiatric comorbidities [14], a psychiatrist (Y.C. Shin, one of the co-first authors) diagnosed all of the patients with GD. We enrolled subjects who scored at least 5 on the Korean version of the South Oaks Gambling Screen (K-SOGS) [15].

There were 23, of the initial 824, cases that were excluded from the study because the family members sought counseling in lieu of the person who gambled. According to the exclusion criteria, additionally 11 participants were excluded due to lifetime diagnosis of a psychotic disorder, mental retardation, and substance use disorder, except for alcohol and nicotine dependence. None of the participants were provided with a therapist’s recommendation to discontinue treatment due to improvement, given that “improvement” is regarded as temporary in GD, and all of the patients were advised to continue treatment throughout their lifetime. However, an additional 32 patients were excluded from the current study because they terminated treatment in this outpatient GD clinic upon agreement with a therapist. Of these patients, 20 were referred to gamblers anonymous (GA) or other outpatient clinics due to the far travel distance to the study clinic. The remaining 12 of the 32 patients who terminated treatment at the clinic were referred to inpatient clinics, or other institutes, because of severe recurrence including the increase of debt or gambling severity. Finally, a total of 758 participants were included in this study. The study protocol was approved by the Ethics Committee of the Kangbuk Samsung Hospital. All of the study subjects provided informed consent prior to participation.

### Intervention

The GD clinic provided individual psychotherapy (PT), pharmacotherapy, and group CBT. The patients simultaneously received distinct therapeutic alternatives, such as GA, if they chose to participate.

Individual PT was based on CBT and motivational enhancement therapy, and lasted for 15–20 min in the outpatient clinic. Regular follow-up was recommended, regardless of whether other types of treatment were utilized [16]. The duration of an individual PT session cannot be longer because the Korean National Health Insurance System pays one CBT fee irrespective of the experts’ work experience (approximately 25 USD for 30 min of CBT with a psychiatrist) [17].

The patients received medication based on their symptoms and clinical presentation. The patients received anticraving drugs for cravings to gamble, and antidepressants to cope with anxiety or depressed mood. To compare the effect of each pharmacotherapy, the psychotropic drugs were classified as anticraving agents (naltrexone, acamprosate) and antidepressants (selective serotonin reuptake inhibitors—escitalopram, paroxetine, sertraline; serotonin–norepinephrine reuptake inhibitors—venlafaxine, milnacipran; norepinephrine–dopamine reuptake inhibitors—bupropion). The ‘no-pharmacotherapy’ group was not administered any major psychotropic prescription drug during the treatment period. Augmented benzodiazepines (as needed) were permitted and not considered in the data analysis. The patients whose main therapy was antipsychotics or mood stabilizers were excluded from the analyses because we assumed that they had an additional major diagnosis.

**Fig. 1:**
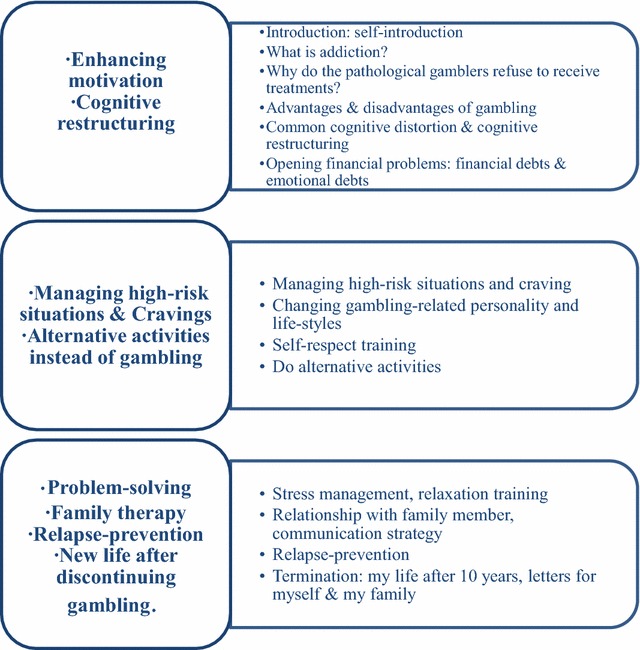
Eight-session group CBT for GD

The group CBT for GD was composed of weekly 2-h sessions, for 8 weeks [18, 19]. All of the group CBT sessions were conducted by a single psychiatrist (Y.C. Shin) three or four times per year from 2004 to 2012, except for the two groups in 2006 (by S.W. Choi). The group CBT included conventional therapeutic elements from CBT, such as psychoeducation, cognitive restructuring, and decisional balance. Moreover, therapeutic skills from motivational enhancement and logotherapy, including problem solving in other areas of life and the meaning of gambling in life, were combined in the treatment. Additional details on the CBT are shown in Fig. 1.

### Measurements

Treatment maintenance duration was evaluated by chart review. If a patient did not visit the GD clinic (unless according to an agreement with a therapist), the patient was regarded as a dropout. For the participants who had not dropped out, the treatment maintenance duration was calculated from the first visit to the day of the last visit before the chart review.

The characteristics of the participants’ gambling behavior were also evaluated at the first visit. The GD patients had a tendency to distort or minimize their debt, due to their gambling behavior [20]. If there was a lack of consensus between a patient and an accompanied family member’s report, then we chose the higher value. The classification of main gambling type was based on the following question: “What type of gambling has most disturbed your life and resulted in the greatest loss of money?” We classified gambling according to structural game characteristics [21]. “Strategic gambling” was defined as gambling activities for which the outcome is believed to be the result of the players’ skill or analysis, such as Poker or Go-stop. “Nonstrategic gambling” was defined as the gambling activities for which the outcome is generally believed to be a result of random probability, such as slot machines, roulette, or lotteries [16].

To evaluate the severity of gambling, the Korean version of the Gambling Symptom Assessment Scale (GSAS) was used [22]. Each item of the GSAS is scored on a 5-point scale from 0 (no symptoms) to 4 (extreme symptoms). The total score ranges from 0 to 48. The Beck Depression Inventory (BDI) and Beck Anxiety Inventory (BAI) were administered to assess comorbid depressive and anxiety symptoms, respectively [23].

### Statistical analysis

To compare the distribution and frequency of the categorical variables, Pearson’s Chi-square test was used. The differences of scale scores and other continuous variables according to intervention were analyzed with independent *t* tests and one-way analysis of variance (ANOVA). Analysis of covariance (ANCOVA) was conducted with BDI, GSAS, and age as the covariates to analyze the total treatment maintenance duration among the three groups and examine the main and interactive effects of other demographic variables. To analyze the independent factors that were associated with the follow-up duration, a generalized linear model was used to adjust each confounding factor. All the analyses were performed using PASW Statistics 17.0 software (SPSS Inc., Chicago, IL, USA), with the cut-off for statistical significance set at p < 0.05.

## Results

### Demographic and clinical characteristics of the participants

Of the 758 subjects, 717 participants (94.59 %) were male. The participants had a mean age of 39.60 ± 9.94 years. The mean age at the onset of GD was 29.84 ± 8.39 years. Of the subjects, 391 (51.58 %) had graduated college or university. More than half of the participants (61.6 %) had a full-time job for more than half of their adult life, and 157 participants (20.8 %) never had a job during their lifetime. Of the patients, 469 (61.87 %) were married.

All of the 758 patients had debt due to GD. Gambling debt at the first visit was 119,897 ± 360,259 US dollars (exchanged from Korean Won). The mean GSAS and K-SOGS scores were 15.56 and 16.27, respectively. The greatest amount of money that each participant had ever won at one time by gambling, a so-called ‘big win’ [24], was 166,471 ± 962,448 US dollars, on average. A total of 150 subjects (19.7 %) gambled online. Of the gambling types, strategic gambling was most preferred (51.6 %), followed by nonstrategic gambling (17.4 %) and mixed (31.0 %). A considerable portion of the subjects (n = 555, 72.9 %) had a psychiatric comorbidity. Depression (9.7 %) was the most prevalent comorbidity, followed by alcohol-related disorders (8.5 %) and bipolar disorder (2.1 %).

### Treatment maintenance duration and pharmacotherapy

The mean treatment maintenance duration was 8.32 ± 16.21 months. Forty-eight patients (6.3 %) did not drop out, with a mean treatment length of 36.43 ± 31.28 months. Of the subjects, 129 (17.01 %) continued to visit the GD clinic for longer than a year, with a follow-up duration of 26.47 ± 24.48 months.

The treatment duration was compared according to the accompanying pharmacotherapy. A total of 477 subjects (62.92 %) composed the no-pharmacotherapy group. The proportion of dropout was significantly lower among the patients who were treated with pharmacotherapy, despite their greater severity of symptoms (F = 8.88, p = 0.003). The treatment maintenance duration was longer in the pharmacotherapy group (t = 5.58, p < 0.001); however, this result had only marginal significance after adjusting for age, depression, and GD severity (F = 3.64, p = 0.057) (Table 1).

**Table 1:** Clinical characteristics of subjects (n = 758)

	No-pharmacotherapy (n = 477)	Pharmacotherapy (n = 281)	Statistical value	p
Demographic characteristics				
Sex (male %)	450 (94.5)	267 (95.0)	0.85^a^	0.388
Age	40.40 ± 10.27	38.24 ± 9.23	2.98^b^	0.003**
Age of onset	30.74 ± 8.92	28.29 ± 7.15	8.10^b^	<0.001**
With partner (or married)	307 (63.1)	166 (59.1)	2.11^a^	0.162
Education (years)	14.14 ± 2.58	13.93 ± 2.54	1.06^b^	0.289
Religion	196 (44.2)	127 (45.2)	0.91^a^	0.350
Gambling characteristics				
Type of main gambling				
Strategic	245 (51.4)	146 (52.0)	2.21^a^	0.332
Nonstrategic	90 (18.9)	42 (14.9)		
Mixed	142 (29.8)	93 (33.1)		
Gambling debt at the first visit ($)	130,470 ± 434,212	102,872 ± 187,295	0.96^b^	0.338
Big win ($)	250,692 ± 1,241,680	44,351 ± 128,800	1.77^b^	0.078
BDI	10.82 ± 10.66	16.89 ± 11.07	−5.26^b^	<0.001**
BAI	16.75 ± 9.68	22.24 ± 9.96	−5.27^b^	<0.001**
GSAS	12.88 ± 9.59	19.01 ± 11.55	−5.36^b^	<0.001**
Intervention				
Group CBT	68 (14.3)	53 (18.9)	2.70^a^	0.102
Participation in GA	106 (25.4)	80 (28.5)	3.13^a^	0.091
Follow-up duration (months)	5.53 ± 12.37	13.03 ± 20.35	−5.58	<0.001**

*BDI* Beck Depression Inventory, *BAI* Beck Anxiety Inventory, *GSAS* Gambling Symptom Assessment Scale, *CBT* cognitive behavioral therapy, *GA* gamblers anonymous

^a^Fischer’s exact test (or Chi-square test): Data are shown as the number of patients (%), statistical value = Pearson’s Chi square

^b^Independent *t* test: Data are shown as mean ± standard deviation, statistical value = *t*

** p < 0.01

1 USD ≒ 1000 won

The patients who took medication were classified into either the antidepressant group (n = 104) or the anticraving agent group (n = 177). Nine subjects were prescribed acamprosate, with a mean dosage of 703.00 ± 260.32 mg/day, and 168 subjects were prescribed naltrexone, with a mean dosage of 36.75 ± 16.62 mg/day. The follow-up duration was 10.37 ± 14.61 months for the anticraving agent group and 17.56 ± 26.99 months for the antidepressant group. The follow-up duration differed between the two pharmacotherapy groups (t = −2.51, p = 0.013), but this difference was not significant after adjusting for age, BDI, and GSAS (F = 1.83, p = 0.161) (Table 2).

**Table 2:** Clinical characteristics according to pharmacotherapy

	No-pharmacotherapy (n = 477)	Anticraving agent (n = 177)	Antidepressants (n = 104)	Statistical value	p
Sex (male %)	450 (94.5)	175 (98.9)	92 (91.1)	9.20^a^	0.010
Age	40.40 ± 10.27	37.45 ± 8.04	39.58 ± 10.86	5.73^b^	0.003**
Age of onset	30.74 ± 8.92	27.87 ± 6.19	29.00 ± 8.56	8.10^b^	<0.001**
With partner (or married)	307 (63.1)	113 (64.8)	53 (51)	6.52^a^	0.038
BDI	10.82 ± 10.66	15.00 ± 10.23	20.67 ± 11.81	6.94^b^	0.002**
BAI	16.75 ± 9.68	20.09 ± 9.24	26.60 ± 10.00	9.72^b^	<0.001**
GSAS	12.88 ± 9.59	20.07 ± 11.83	16.88 ± 10.77	3.01^b^	0.056
Group CBT	68 (14.3)	39 (22)	14 (13.6)	6.23^a^	0.044
Participation in GA	106 (25.4)	54 (34.4)	26 (27.4)	4.59^a^	0.101
Follow-up duration (months)	5.53 ± 12.37	10.37 ± 14.61	17.56 ± 26.99	27.19	<0.001**

*BDI* Beck Depression Inventory, *BAI* Beck Anxiety Inventory, *GSAS* Gambling Symptom Assessment Scale, *CBT* cognitive behavioral therapy, *GA* gamblers anonymous

^a^Pearson’s Chi-square test: Data are shown as the number of patients (%), statistical value = Pearson’s Chi square

^b^One-way analysis of variance (ANOVA): data are shown as mean ± standard deviation, statistical value = *F*

** p < 0.01

A generalized linear model showed the factors that were associated with treatment duration in all GD patients. Sex, age, age of onset, religion, marital status, and other demographic factors were not associated with treatment duration. However, participation in group CBT and antidepressant prescriptions were related to longer treatment duration (Table 3).

**Table 3:** Factors associated with treatment maintenance duration

Parameter	SE	95 % CI		Hypothesis test	
		Lower	Upper	Wald Chi square	Statistical value
Female	5.1326	−6.855	13.264	0.390	0.532
Religion	1.5804	−1.528	4.667	0.986	0.321
Age of onset	0.1086	−.272	0.154	0.295	0.587
BDI	0.0817	−.136	0.184	0.087	0.768
GSAS total	0.0842	−.221	0.109	0.442	0.506
Group CBT	2.0324	10.724	18.691	52.364	<001*
Participation in GA	1.7992	−4.657	2.396	0.395	0.530
Anticraving agent	1.8385	−1.585	5.622	1.205	0.272
Antidepressant	2.3388	1.561	10.729	6.903	0.009*

*BDI* Beck Depression Inventory, *GSAS* Gambling Symptom Assessment Scale, *CBT* cognitive behavioral therapy, *GA* gamblers anonymous

Generalized linear model, * p < 0.05

### Combined effect of pharmacotherapy and group CBT

**Fig. 2:**
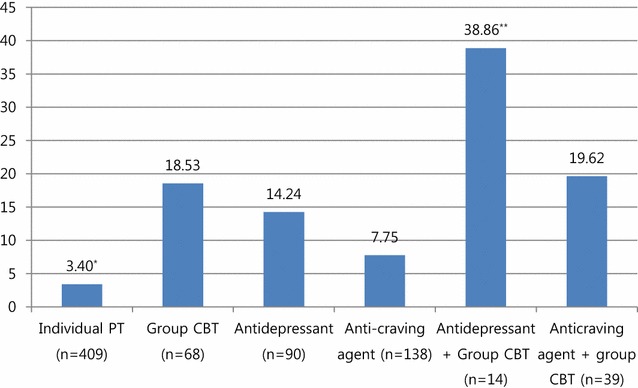
Combined effect of pharmacotherapy and group CBT. All of the treatment groups received individual psychotherapy. Mean follow-up months. *Significantly shorter than all other interventions (p < 0.001). **Significantly longer than all other interventions (p < 0.001)

We also compared the treatment maintenance duration according to the interventions. The mean number of months of follow-up differed across the interventions (F = 35.79, p < 0.001). The patients who received individual PT (n = 409) displayed a significantly smaller number of follow-up months than did all the other intervention groups (3.39 ± 7.51 months), even according to the post hoc analysis (p = 0.03 for the comparison with the anticraving agent group; p < 0.001 for the comparison with the other groups). However, those who received combined group CBT and antidepressant treatment for GD demonstrated the longest follow-up period (38.86 ± 41.77 months) compared with all the other intervention groups (p < 0.001) (Fig. 2).

Additionally, we classified the patients into the following four groups regardless of the type of medication they were prescribed: (1) individual PT (n = 409), (2) group CBT (n = 68), (3) pharmacotherapy (n = 228), and (4) combined group CBT and pharmacotherapy (n = 53). There was a significant difference in follow-up duration among the four groups (F = 48.41, p < 0.001). This result was preserved after adjusting for confounding factors such as age, BDI, and GSAS (F = 27.41, p < 0.001). However, combined group CBT and pharmacotherapy (24.70 ± 28.37 months) was superior to pharmacotherapy only (10.32 ± 16.95 months, F = 26.16, p < 0.001), but not superior to group CBT only in terms of the number of follow-up months (18.53 ± 23.39 months, F = 0.514, p = 0.416).

## Discussion

The current data from 758 Korean GD patients showed that participation in group CBT and medication was associated with longer treatment duration without severe recurrence in an outpatient GD clinic. Antidepressants independently influenced longer maintenance of treatment. Neither demographic factors nor the severity and characteristics of GD were associated with dropout.

To the best of our knowledge, this study is the first to compare treatment duration and dropout according to treatment modality in a clinical setting. GD is a biopsychological disorder that may be attenuated if patients adhere to medication [25]. The current data suggest the importance of appropriate interventions, including combined psychological and biological intervention [26]. Although previous researchers independently demonstrated the effect of pharmacotherapy and group CBT on GD, the current study is the first to investigate the combined effect on treatment adherence [27–29]. As a single psychiatrist gathered the current data over the course of 10 years, the data are free from diversity in the practice and skill of the therapist.

The structure of medical service in South Korea should be considered in order to understand the limited influence of individual PT. This result is in line with the study that compared individual and group CBT [30]. Due to the low medical cost defined by the Korean National Medical Insurance System, Korean psychiatrists manage 28.7 patients per day in the outpatient clinics of university hospitals [31]. Thus, ‘individual PT’ for approximately 15–30 min in outpatient clinic did not enhance the treatment adherence of GD patients. On the other hand, participation in group CBT for 16 h over the course of 8 weeks, including family therapy during the last session, requires the family support that is necessary for successful treatment. Participation in group CBT is also considered to provide psychological support from significant others [32]. We cautiously assumed that if individual PT was performed as intensively as group CBT, then the outcomes of the treatment would be different. The current data do not suggest that group CBT is better than individual PT, but show that intensive treatment with various modalities can enhance treatment adherence. Both group CBT and pharmacotherapy were superior to individual PT in terms of patients’ maintenance of therapeutic alliance.

The association between antidepressant prescription and a lower rate of dropout does not necessarily indicate that antidepressants might be more effective than anticraving agents or other pharmacological interventions for GD patients. If we considered the severity of GD and depression, treatment duration did not differ according to the class of medication. The current data are in agreement with previous research that found no significant difference in the outcome according to the use of antidepressants, anticraving agents, and mood stabilizers to manage GD [33]. Patients who are prescribed antidepressants, such as an emotionally vulnerable group, might be more adherent to treatment than behaviorally conditioned action gamblers [34]. Likewise, those with a mood disorder, or alcohol-dependent patients with depressive symptoms, might have an increased willingness to receive treatment in a clinic.

This study was subject to several limitations. First, dropout does not necessarily indicate the termination of treatment. Although the Kangbuk Samsung Hospital has the largest GD outpatient clinic in South Korea, some patients may have visited a different clinic. Although the patients who were referred to GA were excluded in this study, some patients might have voluntarily registered for GA. Second, this study did not consider natural recovery from addiction [35]. However, the traditional disease model of addiction regards addiction as progressive and irreversible; thus, recovery is difficult without continuous treatment [36]. Third, 94.59 % of the participants were male, which may reduce the generalizability of the present results to female gambling disorder patients. Fourth, the data were derived from 10-year retrospective data; thus, the intervention was not accurately regulated. Specifically, the type and dose of antidepressants were not controlled. Additionally, augmented benzodiazepines were permitted, but not controlled and considered in the data analysis. Furthermore, the GSAS was not measured regularly; thus, various outcomes could not be evaluated. Although remission is not considered due to the high recurrence rate and life-long course of GD, the regular measurement of treatment outcomes, such as GSAS, may aid in the assessment of the outcomes of each treatment. Given that the current research was not planned at the beginning of the study period, individual PT was not structured and objectively observed by other physicians. However, this is also a strength of the study, which showed 10 years of naturalistic data in a real clinical setting.

## Conclusions

This study is the first to examine the clinical characteristics and interventions that affect increased treatment duration in GD patients. Group CBT and any type of medication were associated with a lower rate of dropout. Combined group CBT and antidepressants was the most effective intervention in maintaining the GD patients’ treatment. The current results suggest that multiple treatment approaches are needed to enhance the patients’ motivation and decrease the dropout rate.

## Abbreviations

GD: gambling disorder
CBT: cognitive behavioral therapy
DSM-IV: Diagnostic and Statistical Manual of Mental Disorders, fourth edition
SCID-I: Structured Clinical Interview for DSM-IV Axis I disorders
K-SOGS: Korean version of the South Oaks gambling screen
GA: gamblers anonymous
PT: psychotherapy
GSAS: Gambling Symptom Assessment Scale
BDI: Beck Depression Inventory
BAI: Beck Anxiety Inventory
ANOVA: analysis of variance
ANCOVA: analysis of covariance

## References

[1] Petry NM (2012). Pathological gambling: etiology, comorbidity, and treatment.

[2] American Psychiatric Association (2013). Diagnostic and statistical manual of mental disorders: DSM-5.

[3] Soberay AD, Grimsley P, Faragher JM, Barbash M, Berger B (2014). Stages of change, clinical presentation, retention, and treatment outcomes in treatment-seeking outpatient problem gambling clients. Psychol Addict Behav.

[4] Egorov AI (2014). Modern treatment approaches to gambling. Zhurnal Nevrol Psikhiatrii Im SS Korsakova Minist Zdr Meditsinskoi Promyshlennosti Ross Fed Vserossiiskoe Obshchestvo Nevrol Vserossiiskoe Obshchestvo Psikhiatrov.

[5] Yip SW, Potenza MN (2014). Treatment of gambling disorders. Curr Treat Options Psychiatry..

[6] Grant JE, Odlaug BL, Schreiber LRN (2014). Pharmacological treatments in pathological gambling. Br J Clin Pharmacol.

[7] Leblond J, Ladouceur R, Blaszczynski A (2003). Which pathological gamblers will complete treatment?. Br J Clin Psychol.

[8] Blanco C, Petkova E, Ibáñez A, Sáiz-Ruiz J (2002). A pilot placebo-controlled study of fluvoxamine for pathological gambling. Ann Clin Psychiatry.

[9] Grant JE, Kim SW, Kuskowski M (2004). Retrospective review of treatment retention in pathological gambling. Compr Psychiatry.

[10] Knezevic B, Ledgerwood DM (2012). Gambling severity, impulsivity, and psychopathology: comparison of treatment- and community-recruited pathological gamblers. Am J Addict..

[11] Rosenberg O, Dinur LK, Dannon PN (2013). Four-year follow-up study of pharmacological treatment in pathological gamblers. Clin Neuropharmacol.

[12] Lupi M, Martinotti G, Acciavatti T, Pettorruso M, Brunetti M, Santacroce R (2014). Pharmacological treatments in gambling disorder: a qualitative review. BioMed Res Int..

[13] Dieterich M, Irving CB, Park B, Marshall M (2010). Intensive case management for severe mental illness. Cochrane Database Syst Rev.

[14] American Psychiatric Association (2000). Diagnostic and statistical manual of mental disorders, text revision.

[15] Choi WC, Kim KB, Oh DY, Lee TK (2001). A preliminary study on standardization of korean form of south oaks gambling screening. J Korean Acad Addict Psychiatry..

[16] Shin YC, Choi SW, Ha J, Mok JY, Lim SW, Choi JS (2014). Age of pathological gambling onset: clinical and treatment-related features. J Addict Med..

[17] Mathauer I, Xu K, Carrin G, Evans DB. An analysis of the health financing system of the Republic of Korea and options to strengthen health financing performance. Geneva World Health Organization. 2009. http://cdrwww.who.int/entity/health_financing/documents/hsfr_e_09-korea.pdf. Accessed 24 Jul 2014.

[18] Ha J, Shin YC (2013). Treatment of pathological gambling. J Korean Acad Addict Psychiatry.

[19] Shin YC. Treatment experiences of pathological gambling in Korea. 2014. http://cinpspecialcongress.com/program.php. Accessed 16 Jul 2014.

[20] Potenza MN (2004). Pathological gambling: a clinical guide to treatment.

[21] Ladouceur R, Sévigny S (2005). Structural characteristics of video lotteries: effects of a stopping device on illusion of control and gambling persistence. J Gambl Stud.

[22] Kim HJ, Kim JH, Shin YC, Shin HC, Grant JE, Lee TK (2005). The reliability and validity of the Korean translation of the Gambling Symptom Assessment Scale (KG-SAS). J Korean Neuropsychiatr Assoc.

[23] Sung HM, Kim JB, Park YN, Bai DS, Lee SH, Ahn HN (2008). A study on the reliability and the validity of Korean version of the Beck depression inventory-II (BDI-II). J Korean Soc Biol Ther Psychiatry.

[24] Weatherly JN, Sauter JM, King BM (2004). The “big win” and resistance to extinction when gambling. J Psychol.

[25] Rosenberg O, Dinur LK, Dannon PN (2013). Four-year follow-up study of pharmacological treatment in pathological gamblers. Clin Neuropharmacol.

[26] Iancu I, Lowengrub K, Dembinsky Y, Kotler M, Dannon PN (2008). Pathological gambling: an update on neuropathophysiology and pharmacotherapy. CNS Drugs.

[27] Toneatto T, Brands B, Selby P (2009). A randomized, double-blind, placebo-controlled trial of naltrexone in the treatment of concurrent alcohol use disorder and pathological gambling. Am J Addict.

[28] Grant JE, Kim SW, Hartman BK (2008). A double-blind, placebo-controlled study of the opiate antagonist naltrexone in the treatment of pathological gambling urges. J Clin Psychiatry.

[29] Grant JE, Potenza MN (2006). Escitalopram treatment of pathological gambling with co-occurring anxiety: an open-label pilot study with double-blind discontinuation. Int Clin Psychopharmacol.

[30] Oei TPS, Raylu N, Casey LM (2010). Effectiveness of group and individual formats of a combined motivational interviewing and cognitive behavioral treatment program for problem gambling: a randomized controlled trial. Behav Cogn Psychother.

[31] Korean Health Insurance Review and Assessment Service. http://www.hira.or.kr/main.do (2014). Accessed 29 Jul 2014.

[32] Wood TE, Englander-Golden P, Golden DE, Pillai VK (2010). Improving addictions treatment outcomes by empowering self and others. Int J Ment Health Nurs..

[33] Pallesen S, Molde H, Arnestad HM, Laberg JC, Skutle A, Iversen E (2007). Outcome of pharmacological treatments of pathological gambling: a review and meta-analysis. J Clin Psychopharmacol.

[34] Lobo DSS, Quilty LC, Martins SS, Tavares H, Vallada H, Kennedy JL (2014). Pathological gambling subtypes: a comparison of treatment-seeking and non-treatment-seeking samples from Brazil and Canada. Addict Behav.

[35] Kushnir V, Cunningham JA, Hodgins DC (2013). A prospective natural history study of quitting or reducing gambling with or without treatment: protocol. JMIR Res Protoc.

[36] Klingemann H, Carter-Sobell L (2007). Promoting self-change from addictive behaviors: practical implications for policy, prevention, and treatment.

